# Correlations of CYP2C9∗3/CYP2D6∗10/CYP3A5∗3 gene polymorphisms with efficacy of etanercept treatment for patients with ankylosing spondylitis: A case–control study

**DOI:** 10.1097/MD.0000000000005993

**Published:** 2017-03-03

**Authors:** Yuan-Yuan Chen

**Affiliations:** Department of Rheumatism and Immunology, Affiliated Hospital of Nantong University, Nantong, China.

**Keywords:** ankylosing spondylitis, CYP2C9^∗^3, CYP2D6^∗^10, CYP3A5^∗^3, efficacy, etanercept, gene polymorphism

## Abstract

**Background:**

The tumor necrosis factor alpha (TNF-α) inhibitor etanercept has been proven to be effective in the treatment of ankylosing spondylitis (AS), while genetic polymorphism may affect drug metabolism or drug receptor, resulting in interindividual variability in drug disposition and efficacy. The purpose of this study is to investigate the correlations between CYP2C9∗3/CYP2D6∗10/CYP3A5∗3 gene polymorphisms and the efficacy of etanercept treatment for patients with AS.

**Methods:**

From March 2012 to June 2015, 312 AS patients (174 males and 138 females, mean age: 35.2 ± 5.83 years) from 18 to 56 years old were enrolled in this study. Polymerase chain reaction-restriction fragment length polymorphism was applied to detect the allele and genotype frequencies of CYP2C9^∗^3, CYP2D6^∗^10, and CYP3A5^∗^3 gene polymorphisms. The joint swelling score, erythrocyte sedimentation rate (ESR), and C-reactive protein (CRP) level of AS patients were compared before and after 24-week etanercept treatment. Assessment in Ankylosing Spondylitis (ASAS) and bath ankylosing spondylitis disease activity index (BASDAI) scores were recorded to assess the efficacy of etanercept treatment.

**Results:**

The AS patients with wild-type ^∗^1/^∗^1 and heterozygous ^∗^1/^∗^3 genotypes of CYP2C9^∗^3 polymorphism accounted for 93.59% and 6.41%, respectively, without ^∗^3/^∗^3 genotype. The AS patients with wild-type CC, heterozygous CT, and mutation homozygous TT genotypes of CYP2D6^∗^10 polymorphism accounted for 19.23%, 39.10%, and 41.67%, respectively. The AS patients with wild-type ^∗^1/^∗^1, heterozygous ^∗^1/^∗^3, and mutation homozygous ^∗^3/^∗^3 genotypes of CYP3A5^∗^3 polymorphism accounted for 7.69%, 36.22%, and 56.09%, respectively. After 24-week treatment, AS patients with wild-type ^∗^1/^∗^1 genotype of CYP2C9^∗^3, CC genotype of CYP2D6^∗^10, and ^∗^3/^∗^3 genotype of CYP3A5^∗^3 polymorphisms had lower joint swelling score, ESR, and CRP level. The joint swelling score, ESR, and CRP levels were significantly lower in the patients with CC genotype of CYP2D6^∗^10 polymorphism than in CT and TT genotype patients, and they were lower in patients with ^∗^3/^∗^3 genotype of CYP3A5^∗^3 polymorphism compared to those with ^∗^1/^∗^1 and ^∗^1/^∗^3 genotypes. Average visual analog scale scores of 4 ASAS20 indexes were decreased after treatment. The patients with CC genotype of CYP2D6^∗^10 polymorphism and ^∗^3/^∗^3 genotype of CYP3A5^∗^3 polymorphism exhibited higher scores of >ASAS20, >BASDAI50%, and effective rate.

**Conclusion:**

Our results indicate that CC genotype of CYP2D6^∗^10 polymorphism and ^∗^3^∗^3 genotype of CYP3A5^∗^3 polymorphism are correlated with the efficacy of etanercept treatment for AS patients.

## 1 Introduction

Ankylosing spondylitis (AS), a chronic inflammatory disease, is a kind of spondyloarthropathy, damaging the spine and sacroiliac joints with debilitating pain. The flexibility and mobility of the spine may be reduced and finally lost.^[1]^ After the primary onset of symptoms, it can take 5 to 10 years to be accurately diagnosed as AS.^[2]^ AS affects 0.1% to 1.4% of the general population, and man suffers from this disease predominantly, varying from 20 to 40 years, with critical loss of work productivity and sharp decline of their quality of life.^[3]^ According to a recent study, although current drug treatments have improved the symptoms of AS patients to some degree, they still remain unsatisfactory; however, the tumor necrosis factor alpha (TNF-α) inhibitor etanercept has been proven to be effective in the treatment of AS.^[4]^ Etanercept is used primarily for the therapy of rheumatoid arthritis (RA) in 1998.^[5]^ Composed of a recombinant human TNF receptor, etanercept is a fusion protein that blocks the cytokine TNF-α and sometimes the TNF transmembrane receptor (rTNF), decreasing chronic inflammatory processes.^[6]^ Pharmacogenetics and pharmacogenomics studies have confirmed that genetic polymorphism may have an impact on drug metabolism, drug targets, or drug receptor, resulting in interindividual variability in drug disposition and efficacy.^[7,8]^

Recent studies have demonstrated that variants in cytochrome P-450 (CYP) genes can result in differences in the expression and function of their relevant encoding enzymes, thus affecting individual's response to drug.^[9,10]^ Previous evidence demonstrated that the biotransformation and elimination of warfarin are mainly caused by oxidation through cytochrome CYP2C9. At present, 2 variant alleles of this cytochrome that can lower enzymatic activity in vitro have been recognized: CYP2C9^∗^2 (Arg144Cys) as well as CYP2C9^∗^3 (Ile359Leu).^[11]^ The CYP2D6 polymorphism is a commonly found mutant allele in Asians. CYP2D6^∗^1 is carried by 37.9% of the whole Chinese population; while the variant CYP2D6^∗^10 becomes the most usual allele, accounting for 51.3%.^[12]^ Furthermore, CYP3A4 and CYP3A5 affect over 60% of licensed drugs. Thus, individual single nucleotide polymorphisms variants with CYP3A4 and CYP3A5 genes may exert influence on the expression of enzyme.^[13]^ CYP3A5^∗^3, most common in CYP3A5, can change the CYP3A5 mRNA expression and function of enzyme, which may cause the nonfunctional CYP3A5 protein.^[10]^ And a study also reports the possible involvement of TNF-α in the down-regulation of CYP3A due to TNF-α as one of the potent mediators repressing CYP3A transcription.^[14]^ In the present study, the author investigated the correlations of CYP2C9^∗^3, CYP2D6^∗^10, and CYP3A5^∗^3 gene polymorphisms with the efficacy of etanercept treatment for AS patients.

## 2 Materials and methods

### 2.1 Ethical statement

This study was approved by the Ethics Committee of Affiliated Hospital of Nantong University, and all patients have signed the informed consent. The ethical approval for this study conformed to the standards of the Declaration of Helsinki.^[15]^

### 2.2 Study subjects

From March 2012 to June 2015, a total of 312 AS patients (174 males and 138 females, mean age: 35.2 ± 5.83 years) ages from 18 to 56 years old in Affiliated Hospital of Nantong University were enrolled into our study. The diagnoses of all patients in our study were in line with the modified New York criteria for AS: the visual analog scale (VAS) score of AS ≥30; at least 2 VAS scores of spinal pain, overall evaluation of patients with disease activity and physical function ≥30. Exclusive criteria: patients who had taken hormones with other immunosuppressive drugs in 3 months before the enrollment; patients with cardiovascular and cerebrovascular diseases; patients with history of thrombosis and thromboembolic disease; patients with diabetes, viral hepatitis, cirrhosis, severe liver and kidney dysfunction, severe malnutrition, or thyroid-related disease; patients who were pregnant; patients who have received organ transplantation; and patients with active tuberculosis.

### 2.3 Genomic DNA extraction and genotyping

Peripheral blood (3–5 mL) was collected from all study subjects, and anticoagulated conventional phenol extraction method was conducted with 1% ethylenediaminetetraacetic acid to extract genomic DNA, which was then diluted to a final concentration of 10 ng/μL. Primers for polymerase chain reaction (PCR) were designed by Primer Premier 5.0 software and synthesized by Shanghai Sangon Biological Engineering Company (Shanghai, China). The primer sequences and their length were shown in Table 1. PCR system contains template DNA (10 ng), buffer solution (10× PCR), each primer (15 pmol), dNTPs (0.2 mmol/L), and *Taq* 0.12 U enzyme plus double-distilled water to 15 μL. PCR was performed on GeneAmP PCR system 9700 under amplification condition using touchdown PCR: 94°C for 2 min, 94°C for 30 s, 63°C for 1 min, and 72°C for 1 min; then 94°C for 30 s, 57°C for 1 min, 72°C for 1 min, and 72°C for 7 min. The amplified products were subject to 1.5% agarose gel electrophoresis, and estimation of purity and concentration of the amplified bands were made under the ultraviolet lamp. The PCR amplification products of the 3 genes (each 3 μL) were selected to react with different endonucleases at 37°C for 45 min and 85°C for 15 min. Prism BigDye Terminator (BDT) Cycle Sequencing Kit (ABI Company, Oyster Bay, NY, USA) was employed to add 1 μL reverse primer and 1 μL BDT into purified PCR products.

**Table 1:**
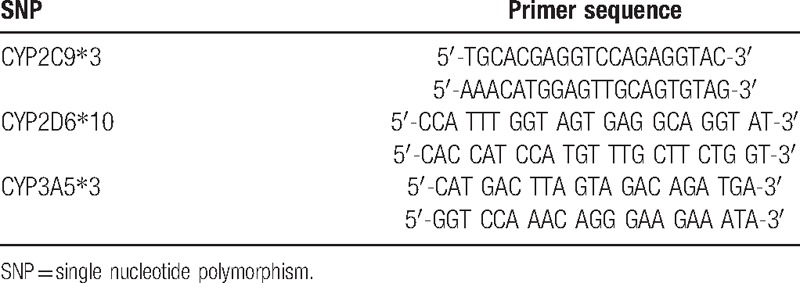
Primer sequences of SNP CYP2C9^∗^3/CYP2D6^∗^10/CYP3A5^∗^3.

### 2.4 Therapeutic regimen and etanercept efficacy assessment

All AS patients were treated with 50 mg etanercept by subcutaneous injection (once a week) for 24 weeks. After that AS patients’ clinic indicators before and after the treatment were compared to assess the efficacy of etanercept.

Joint swelling score was employed as an indicator to compare the degree of circumferential joint swelling before and after the treatment; 0 point, no joint swelling; 1 point, mild joint swelling with obvious bone landmark; 2 points, moderate joint swelling with unobvious bone landmark; and 3 points, severe joint swelling with no bone landmark or with effusion; the scores of all diseased joints were summed to get the final joint swelling score.

C-reactive protein (CRP) level and erythrocyte sedimentation rate (ESR) were measured separately before and after the treatment. Reference ranges of ESR ≤15 mm/h for male and ≤20 mm/h for female; that of CRP ≤ 8 mg.

Assessment in Ankylosing Spondylitis (ASAS) 20 and bath ankylosing spondylitis disease activity index (BASDAI) 50 issued by the international Ankylosing Spondylitis Assessment Study group in 2001 were used as a standard to assess the efficacy of etanercept.

According to ASAS20, the etanercept treatment was regarded efficient when the patients got equal to or over 20% improvements, or at least 1 score in progression, compared with baseline characteristics in any 3 of the following 4 items overall evaluation (VAS); spinal pain (VAS); bath ankylosing spondylitis functional index (BASFI); and spinal inflammation, severity of morning stiffness, and duration of morning stiffness (VAS). The treatment was regarded inefficient when patients failed to get 20% improvement of the above indicators, and no deterioration was observed in comparison to baseline characteristics (ASAS50/70 meant 50% and 70% improvements of the above indicators, respectively, VAS).

According to BASDAI50, the etanercept treatment was regarded efficient when AS patients showed over 50% BASDAI improvement compared with baseline characteristics; the treatment was judged inefficient or moderately efficient when AS patients showed <20% or 20% to 50% BASDAI improvement compared with baseline characteristics, respectively.

### 2.5 Follow-up

Follow-up was conducted by telephone call, outpatient service, and medical clinical records, which lasted for 24 weeks with the deadline in December 2015, and the follow-up rate was 97.4%. During the follow-up, related indexes were recorded, including morning stiffness, functional examination, ESR, CPR, BASDAI score, ASAS20/50/70 score, the proportion of involved circumferential joints, and proportion of abnormal ESR and CRP.

### 2.6 Statistical analysis

SPSS 17.0 was used for data analysis, with *P* < 0.05 as statistically significant. Genotype and allele frequencies were calculated by direct counting method. And the allele and genotype were compared by χ^2^ test. The χ^2^ test and Fisher exact test were performed to analyze improvement ratio and percentage of AS patients. After normal distributions were examined, different genotype carriers were compared with each other on BASDAI score improvement before and after 24-week treatment. The *t* test for normal distribution and rank-sum test for non-normal distribution were performed. Other indexes were presented as mean ± standard deviation.

## 3 Results

### 3.1 Electrophoresis of PCR product

**Figure 1:**
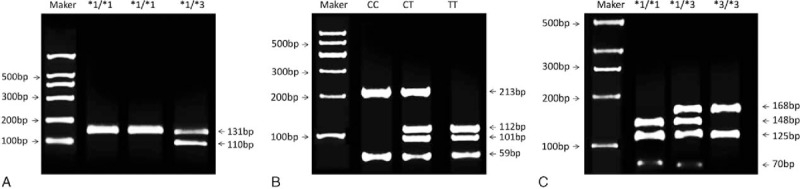
Electrophoretograms of polymerase chain reaction-restriction fragment length polymorphism enzyme-digested products. (A) Electrophoretogram of CYP2C9^∗^3 after *KPn*I enzyme digestion; (B) electrophoretogram of CYP2D6^∗^10 after *HPh*I enzyme digestion; (C) electrophoretogram of CYP3A5^∗^3 after *SsP*I enzyme digestion.

After *KPn*I enzyme digestion, the PCR products of CYP2C9^∗^3 showed the wild-type homozygote ^∗^1/^∗^1 with 1 band 131 bps and mutant heterozygote ^∗^1/^∗^3 with 3 bands 131, 110, and 21 bps (the 21 bps was too small to stay in gel, hence the mutant heterozygotes merely showed 2 bands: 131 and 110 bps). And the mutant homozygote ^∗^3/^∗^3 which showed 110 and 21 bps were not observed in this study (Fig. 1A).

After *HPh*I enzyme digestion, the PCR products of CYP2D6^∗^10 were as follows: the wild-type homozygote CC had 1 *HPh*I recognition site, with 2 fragments of 213 and 59 bps. The mutant homozygote TT had a new enzyme recognition site due to replacement of C with T, with 3 fragments of 112, 101, and 59 bps, while the mutant heterozygote CT had 4 fragments of 213, 112, 101, and 59 bps (Fig. 1B).

After *SsP*I enzyme digestion, the PCR products of CYP3A5^∗^3 were as follows: the wild-type homozygote ^∗^1/^∗^1 had 2 *SsP*I enzyme recognition sites, and the 293 bps was digested into 3 fragments of 148, 125, and 70 bps. The mutant homozygote ^∗^3/^∗^3 merely had 1 *SsP*I enzyme recognition site, and the 293 bps was digested into 2 fragments of 168 and 125 bps. The mutant heterozygote ^∗^1/^∗^3 had 2 *SsP*I enzyme recognition sites, and the product was digested into 4 fragments of 168, 148, 125, and 70 bps (Fig. 1C).

### 3.2 Genotype and allele frequencies of CYP2C9^∗^3/CYP2D6^∗^10/CYP3A5^∗^3 polymorphisms

Among 312 patients with CYP2C9^∗^3 gene, ^∗^1/^∗^1 homozygote accounted for 93.59%, ^∗^1/^∗^3 heterozygote accounted for 6.41%, and there was no ^∗^3/^∗^3 genotype. And the frequency of ^∗^1 allele was 96.79% and ^∗^3 allele was 3.21%. As for CYP2D6^∗^10 polymorphism, CC homozygote accounted for 19.23%, CT heterozygote for 39.10%, and TT homozygote for 41.67%. And the frequency of T allele was 61.22%. With regard to CYP3A5^∗^3 gene polymorphism, 7.69% of patients had ^∗^1/^∗^1 homozygote, 36.22% of patients had ^∗^1/^∗^3 heterozygote, and 56.09% of patients had ^∗^3/^∗^3 homozygote. And the frequency of ^∗^3 allele was 74.20%. All genotypes and allele frequencies were tested by the Hardy–Weinberg genetic equilibrium and met the population genetic equilibrium (Table 2).

**Table 2:**
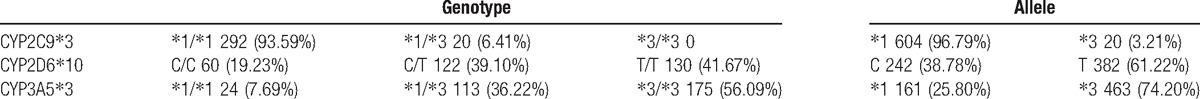
Genotype and allele frequencies of CYP2C9^∗^3/CYP2D6^∗^10/CYP3A5^∗^3 polymorphisms.

### 3.3 Comparison of joint swelling score before and after etanercept treatment for AS patients with different genotypes

After 24-week etanercept treatment, the patients with ^∗^1/^∗^1 genotypes of CYP2C9^∗^3 polymorphism, CC genotype of CYP2D6^∗^10 polymorphism, and ^∗^3/^∗^3 genotypes of CYP3A5^∗^3 polymorphism had lower joint swelling scores than that before the treatment (all *P* < 0.05). The AS patients with CC genotype of CYP2D6^∗^10 showed lower joint swelling scores compared to those with CT and TT genotype; those with ^∗^3/^∗^3 genotypes of CYP3A5^∗^3 had lower joint swelling scores compared to those with ^∗^1/^∗^3 genotype (all *P* < 0.05). However, there was no significant difference in the patients with different genotypes of CYP2C9^∗^3 (all *P* > 0.05) (Table 3).

**Table 3:**
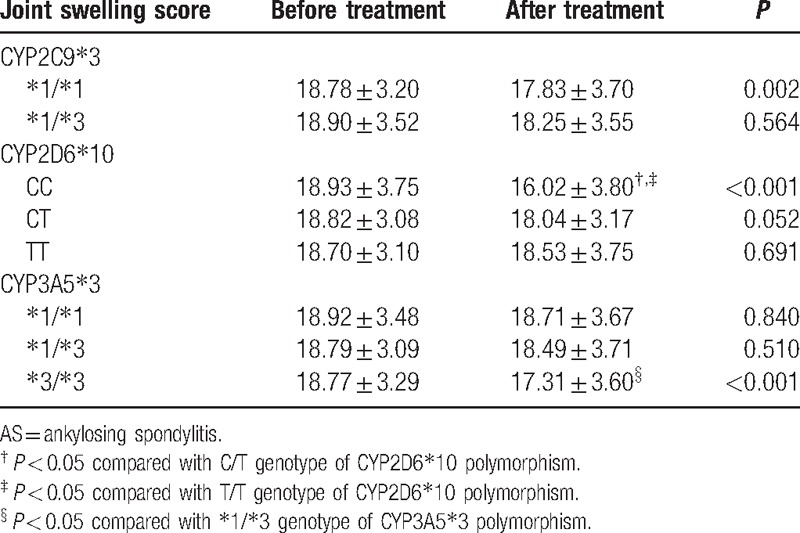
Comparison of joint swelling score before and after etanercept treatment for AS patients with different genotypes.

### 3.4 Comparisons of ESR and CRP level before and after etanercept treatment for AS patients with different genotypes

After 24-week etanercept treatment, AS patients with ^∗^1/^∗^1 genotype of CYP2C9^∗^3 polymorphism, CC genotype of CYP2D6^∗^10 polymorphism, and ^∗^3/^∗^3 genotype of CYP3A5^∗^3 polymorphism exhibited lower ESR and CRP levels (all *P* < 0.05). The ESR and CRP level in the patients with CC genotype of CYP2D6^∗^10 polymorphism were significantly lower than in those with CT and TT genotype (all *P* < 0.05). Also, patients with ^∗^3/^∗^3 genotype of CYP3A5^∗^3 polymorphism showed lower ESR and CRP level compared to those with ^∗^1/^∗^1 and ^∗^1/^∗^3 genotypes (all *P* < 0.05). There were no significant differences in ESR and CRP level of AS patients with different genotypes of CYP2C9^∗^3 before and after treatment (all *P* > 0.05) (Table 4).

**Table 4:**
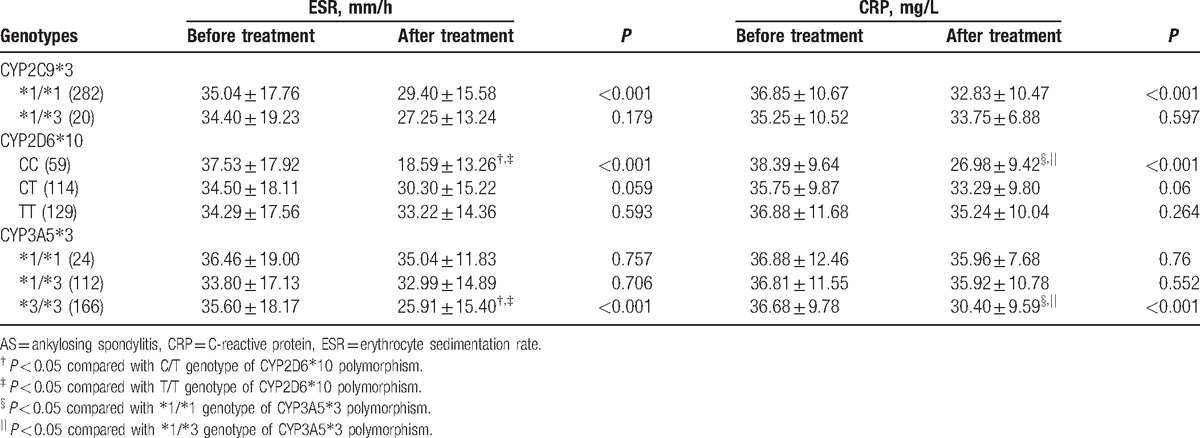
Comparisons of ESR and CRP before and after etanercept treatment for AS patients with different genotypes.

### 3.5 Changes of 4 major items of ASAS20 after etanercept treatment for AS patients

Three hundred twelve patients were treated with etanercept in subcutaneous injection once a week. And among them, there were 302 patients who had 24-week follow-up records. After 24 weeks of the treatment, the average VAS scores of 4 major items of ASAS20, including overall evaluation, spinal pain, BASFI, severity, and duration of morning stiffness, were decreased (all *P* < 0.05) (Table 5).

**Table 5:**
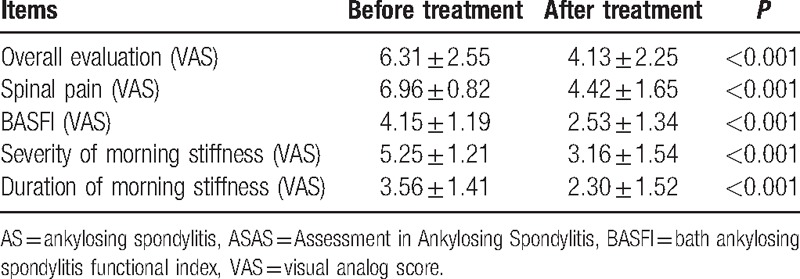
Changes of 4 major items of ASAS20 before and after etanercept treatment for AS patients.

### 3.6 Comparisons of ASAS20/50/70 scores after etanercept treatment for AS patients with different genotypes

After 24 weeks of etanercept treatment, there were no statistical differences in >ASAS20/50/70 scores for patients with CYP2C9^∗^3 polymorphism (all *P* > 0.05). As for AS patients with CYP2D6^∗^10 polymorphism, after 24-week etanercept treatment, higher proportion of patients with CC genotype (98.31%) met 20 < ASAS < 50 standard than those with CT (85.09%) and TT (89.15%) genotypes (both *P* < 0.05); higher proportion of patients with CC genotype (66.10%) met 50 < ASAS < 70 standard than those with CT (17.54%) and TT (9.30%) genotypes (both *P* < 0.05); also, there was more patients with CC genotype (37.29%) met ASAS > 70 standard than those with CT (1.75%) and TT (1.55%) genotypes (both *P* < 0.05). Additionally, after 24-week etanercept treatment, AS patients with ^∗^3/^∗^3 genotype of CYP3A5^∗^3 polymorphism (31.43%) had higher proportion to meet 50 < ASAS < 70 standard than those with ^∗^1/^∗^1 (8.33%) and ^∗^1/^∗^3 (12.50%) genotypes, and higher proportion of AS patients with ^∗^3/^∗^3 (13.86%) genotype of CYP3A5^∗^3 polymorphism met ASAS > 70 standard than those with ^∗^1/^∗^3 (1.79%) genotype (all *P* < 0.05) (Table 6).

**Table 6:**
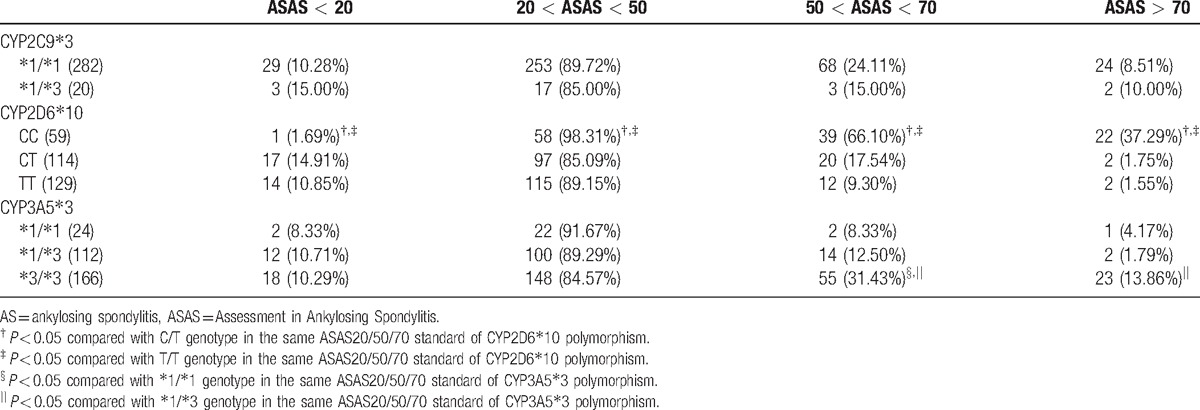
Proportion of AS patients with different genotypes achieving ASAS20/50/70 scores after 24-wk etanercept treatment.

### 3.7 Comparison of BASDAI score before and after etanercept treatment for AS patients with different genotypes

After 24 weeks of etanercept treatment, no improvement of BASDAI score was seen in AS patients with ^∗^1/^∗^1 and ^∗^1/^∗^3 genotypes of CYP2C9^∗^3 polymorphism (both *P* > 0.05). Lower proportion of AS patients with CC genotype of CYP2D6^∗^10 polymorphism (8.47%) met the <BASDAI20% improvement than those with CT (25.44%) and TT (34.11%) genotypes (all *P* < 0.05); however, higher proportion of patients with CC genotype of CYP2D6^∗^10 polymorphism (45.76%) achieved >BASDAI50% improvement than those with TT genotype (27.91%) (*P* < 0.05). As for CYP3A5^∗^3 polymorphism, lower proportion of patients with ^∗^3/^∗^3 genotypes (18.07%) achieved <BASDAI20% than those with ^∗^1/^∗^1 (50.00%) and ^∗^1/^∗^3 (32.14%) genotypes (all *P* < 0.05); whereas higher proportion of patients with ^∗^3/^∗^3 genotypes (43.37%) achieved <BASDAI50% than those with ^∗^1/^∗^1 (25.00%) and ^∗^1/^∗^3 (25.00%) genotypes (all *P* < 0.05) (Table 7).

**Table 7:**
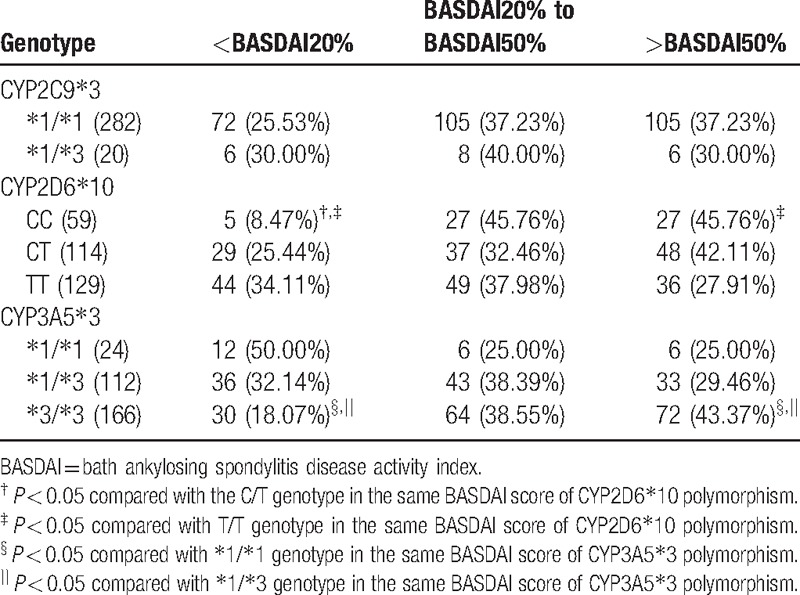
Proportion of patients with different genotypes achieving BASDAI score after 24-wk etanercept treatment.

### 3.8 Efficacy of etanercept in AS patients with different genotypes assessed by ASAS20 and BASDAI50 scores

No statistical difference in etanercept efficacy was observed between patients with ^∗^1/^∗^1 and ^∗^1/^∗^3 genotypes of CYP2C9^∗^3 polymorphism (both *P* > 0.05). A higher effective rate of etanercept was found in patients with CC (89.83%) genotype of CYP2D6^∗^10 compared with those with CT (72.81%) and TT (49.61%) genotypes (both *P* < 0.05). Besides, AS patients with ^∗^3/^∗^3 (80.12%) genotype of CYP3A5^∗^3 showed a higher effective rate of etanercept than those with ^∗^1/^∗^1 (45.83%) and ^∗^1/^∗^3 (50.00%) genotypes (both *P* < 0.05) (Table 8).

**Table 8:**
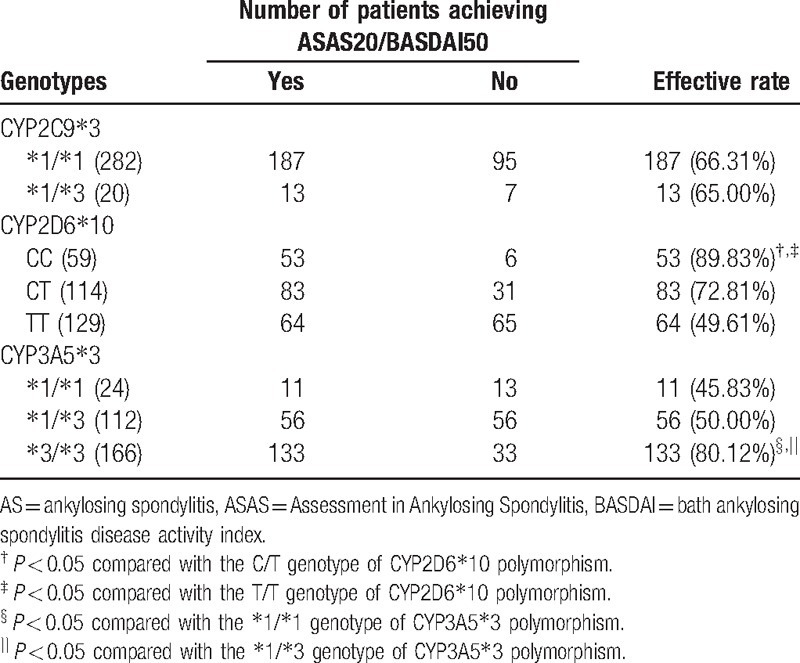
Efficacy of etanercept in AS patients with different genotypes assessed by ASAS20 and BASDAI50 scores.

## 4 Discussion

Etanercept is composed of a recombinant human TNF receptor, a fusion protein that blocks the cytokine TNF-α and sometimes rTNF, decreasing the process of chronic inflammatory.^[6]^ Although etanercept is considered as a widely used treatment for AS, the outcome remains unsatisfactory due to interindividual variability in drug disposition and efficacy. It is well known that CYP enzymes may be involved in drug interactions during treatments for chronic inflammatory rheumatic diseases.^[16,17]^ Hopkins et al have reported that genetic polymorphism of CYP450 enzyme gene (CYP1A2) was linked to increased leflunomide toxicity in the treatment of RA.^[18]^ Soukup et al also demonstrated that genetic polymorphisms of CYP450 enzyme genes (CYP1A2, CYP2C19, and CYP3A4) are involved in leflunomide metabolite activation in the treatment of RA.^[19]^ Feng et al have provided evidence that CYP450 enzymes (CYP2C8 and CYP2J2) overexpression could markedly suppress the activity of TNF-α and TNF-α-induced inflammatory cytokines.^[20,21]^ Furthermore, a genome-wide association study by Brown et al has confirmed that CYP450 enzyme gene polymorphisms may increase susceptibility to AS.^[22,23]^ Therefore, the author hypothesizes that polymorphisms in CYP450 enzyme genes alter the disease contribution of TNF-α which would then influence the efficacy of etanercept treatment.

The results in this study have confirmed that the efficacy of etanercept in the treatment of AS is related to CYP2D6^∗^10 polymorphism and CYP3A5^∗^3 polymorphism. The ^∗^1/^∗^1 genotype of CYP2D6^∗^10 polymorphism and ^∗^3/^∗^3 genotype of CYP3A5^∗^3 polymorphism had higher ASAS20/50/70 and BASDI scores than ^∗^1/^∗^3 and ^∗^3/^∗^3 genotypes of CYP2D6^∗^10 polymorphism and ^∗^1/^∗^1 and ^∗^1/^∗^3 genotypes of CYP3A5^∗^3 polymorphism, respectively. According to Gupta et al, there is indeed a possible association between TNF-α inhibitors (including infliximab, etanercept, adalimumab, and ustekinumab) and CYP450 activity.^[24]^ Many prescribed drugs are metabolized by the indispensable cytochrome P450 enzymes, among which the highly polymorphic CYP2C9, one of the enzymes, accounts for about 20% of the metabolizing of clinical medicines including warfarin.^[25]^ The mutation of CYP3A5 gene can cause the change of CYP3A5 enzyme activity, which may lead to the decrease of the metabolic clearance rate, thus increasing the concentration of plasma drugs, which indicated the increase of efficacy.^[26]^ The mRNA expression and function of enzyme can be changed by CYP3A5^∗^3, which was commonly discovered in CYP3A5, and it is universally acknowledged that the alternative splicing event caused by CYP3A5^∗^3 may bring about nonfunctional CYP3A5 protein decreasing the metabolic efficacy of its substrate.^[10]^ A previous study confirms that CYP3A5 genetic polymorphism is a good predictor in determining daily requirements of immunosuppressive drugs.^[27]^ According to Sandrine et al, many gene polymorphisms, which are correlated with low production of TNF-α, are also correlated with better efficacy of etanercept treatment that can suppress TNF-α expression in RA.^[28]^ Giuseppe et al demonstrated that other gene polymorphisms have been good predictors for response to treatment with TNF-α inhibitors (such as etanercept, infliximab, and golimumab) in AS patients.^[29]^

In summary, our findings indicate that CYP2D6^∗^10 and CYP3A5^∗^3 polymorphisms may serve as biomarkers for response to etanercept treatment in AS. Response to drug therapy is varied due to different gene background. With the help of these variable factors, clinicians can offer patients more reasonable and appropriate prescription, thus avoiding the misuse of drugs and finding potential value of etanercept.
